# Low-dose multi-detector computed tomography for periradicular infiltrations at the cervical and lumbar spine

**DOI:** 10.1038/s41598-022-08162-8

**Published:** 2022-03-12

**Authors:** Karolin J. Paprottka, Karina Kupfer, Vivian Schultz, Meinrad Beer, Claus Zimmer, Thomas Baum, Jan S. Kirschke, Nico Sollmann

**Affiliations:** 1 Department of Diagnostic and Interventional Neuroradiology, School of Medicine, Klinikum rechts der Isar, Technical University of Munich, Munich, Germany; 2 TUM-Neuroimaging Center, Klinikum rechts der Isar, Technical University of Munich, Munich, Germany; 3 Department of Diagnostic and Interventional Radiology, University Hospital Ulm, Ulm, Germany; 4 Department of Radiology and Biomedical Imaging, University of California, San Francisco, CA USA

**Keywords:** Computed tomography, Medical imaging

## Abstract

Periradicular infiltrations are frequently performed in daily neuroradiological routine and are often guided by multi-detector computed tomography (MDCT), thus leading to radiation exposure. The purpose of this study was to evaluate MDCT with low dose (LD) and model-based iterative reconstruction for image-guided periradicular infiltrations at the cervical and lumbosacral spine. We retrospectively analyzed 204 MDCT scans acquired for the purpose of cervical or lumbosacral periradicular interventions, which were either derived from scanning with standard dose (SD; 40 mA and 120 kVp) or LD (20–30 mA and 120 kVp) using a 128-slice MDCT scanner. The SD cases were matched to the LD cases considering sex, age, level of infiltration, presence of spinal instrumentation, and body diameter. All images were reconstructed using model-based iterative image reconstruction and were evaluated by two readers (R1 and R2) using 5- or 3-point Likert scales (score of 1 reflects the best value per category). Furthermore, noise in imaging data was quantitatively measured by the standard deviation (StDev) of muscle tissue. The dose length product (DLP) was statistically significantly lower for LD scans (6.75 ± 6.43 mGy*cm vs. 10.16 ± 7.70 mGy*cm; *p* < 0.01; reduction of 33.5%). Image noise was comparable between LD and SD scans (13.13 ± 3.66 HU vs. 13.37 ± 4.08 HU; *p* = 0.85). Overall image quality was scored as good to very good with only minimal artifacts according to both readers, and determination of the nerve root was possible in almost all patients (LD vs. SD: *p* > 0.05 for all items). This resulted in high confidence for intervention planning as well as periprocedural intervention guidance for both SD and LD scans. The inter-reader agreement was at least substantial (weighted Cohen’s κ ≥ 0.62), except for confidence in intervention planning for LD scans (κ = 0.49). In conclusion, considerable dose reduction for planning and performing periradicular infiltrations with MDCT using model-based iterative image reconstruction is feasible and can be performed without clinically relevant drawbacks regarding image quality or confidence for planning.

## Introduction

Periradicular infiltrations are well known to be an effective symptomatic treatment performed in patients with radiculopathy-associated pain syndromes (therapeutic periradicular infiltrations)—especially in patients with underlying disc prolapse, one of the most frequent causes of radiculopathy^1,2^. Furthermore, periradicular infiltrations can be a good add-on tool to diagnose the distinct site causing symptoms (diagnostic periradicular infiltrations)^3^. Therefore, these procedures are among the most frequently performed exams in daily neuroradiological routine^4–8^. Furthermore, periradicular infiltrations have been implemented as reliable methods to guide pain management, especially among patients in whom conservative approaches such as drug treatment have been exhausted^9,10^.

The procedure of a periradicular infiltration is commonly performed with image guidance to guarantee a precise navigation with respect to the individual anatomy in the area of intervention^11^. As conventional fluoroscopy is still the standard modality in clinical routine, cross-sectional imaging modalities like computed tomography (CT) are receiving more and more acceptance in the interventional routine and are therefore well evaluated in case of safety, technical nuances, and radiation dose parameters. Specifically, multi-detector CT (MDCT) is often used, allowing for initial procedure planning and subsequent surveillance of the procedure. Compared to non-navigated approaches, using interventional CT scanning has shown not only to improve safety as well as precision and pain reduction, but also to reduce the overall treatment duration for periradicular infiltrations^12,13^. Furthermore, interventional procedures at the spine under CT guidance afford the interventionalist several advantages over conventional fluoroscopic methods, as the interventionalist has a direct visualization of the needle tip to target soft tissues during the whole procedure as well as a superior contrast resolution and an improved ability to navigate in case of difficult anatomical situations, such as severe osseous stenosis^14^. Due to these advantages, CT has been used with increased frequency for pain-relieving injections at the spine^15^.

However, the broader use of CT has also raised concerns regarding radiation exposure for patients as well as for the performing interventionalists^16–21^. One of the main disadvantages of CT-guided procedures compared with the conventional fluoroscopy procedure is the risk of a distinctly higher radiation exposure to patients^15^. Therefore, over the last years, effort has been spent on the different possibilities of dose reduction in diagnostic and interventional CT, which can have varying effects on resulting image quality^4,5,22–26^. Approaches to reduce dose during planning CT include different major options. Limiting the *z*-axis to the level of interest, modifying CT acquisition parameters (such as tube current, tube voltage, or acquisition coverage), and tailoring CT parameters to patient body habitus are capable options for reducing exposure^25,27,28^. Specifically, studies regarding CT in adult patients with suspected lumbar disc herniation could show a dose reduction of up to 35% by lowering the tube current without degrading image quality to a relevant extent^29^. Furthermore, a simulation study for systematic evaluation of MDCT with low dose (LD) for planning purposes of lumbosacral periradicular infiltrations showed that virtually lowering the tube current down to 10% of standard dose (SD) may be possible without limitations for intervention planning confidence^30^. However, such drastic dose reductions may not be directly transferred to the ultimate clinical setting as they result from post-hoc simulations in combination with advanced image reconstruction procedures^30^.

Of note, previous work suggested that the main part of the radiation exposure in CT-guided procedures is caused by the periprocedural CT scanning^31^. Moreover, the planning CT acquired before CT-guided interventions contributed significantly to the overall radiation dose^15,32^. Given the advancements in modern image reconstruction algorithms that may enable further radiation dose decreases without relevant restrictions for image quality, it is important to especially consider the planning scans for further optimization regarding radiation exposure. Against this background, in this study we compare planning of cervical or lumbosacral periradicular infiltrations based on MDCT with lowered tube current and model-based iterative image reconstruction against MDCT using SD scanning.

## Material and methods

### Study cohort

We retrospectively reviewed CT-guided periradicular lumbosacral and cervical injections performed with two different dose levels: SD and LD scans for procedure planning and performance of infiltrations. A general adjustment of our institutional CT protocols took place in October 2020. Thus, all LD scans included in this study were acquired between November 2020 and June 2021, while SD scans were derived from the interval of January 2020 to September 2020.

Patients with LD scans were consecutively included if they had MDCT scanning available for a periradicular lumbosacral or cervical infiltration according to clinical indications (to treat radiculopathy-related pain and/or to achieve a selective nerve root block for diagnostic purposes). Patients were identified in the hospital’s institutional digital picture archiving and communication system (PACS). Eligible patient cases with LD scans were matched to patients with SD scans according to sex, age (± 5 years), level of infiltration (± 1 level), presence/absence of spinal instrumentation (metallic hardware causing artifacts and making the access route to the nerve root more demanding), and body diameter (> 20 cm, 20–25 cm, 25–30 cm, and > 30 cm). Patients were excluded if (1) non-diagnostic image quality was present in MDCT data (e.g., due to motion artifacts), (2) the periradicular infiltration (including survey, planning, and procedure scans) was not accomplished (e.g., due to incompliance of the patient and preliminary abortion of the exam), or (3) there were no cases available that fulfilled the matching criteria. Overall, 204 cases were eligible (102 SD scans and 102 LD scans) and considered in this study.

### Multi-detector computed tomography

All scans included were performed with the patient in prone position using the same institutional 128-slice MDCT scanner (Ingenuity Core 128; Philips Healthcare, Best, The Netherlands). After performing the scout of either the cervical or lumbosacral spine, a planning scan of the region to be treated was performed (spot scanning). The acquired scan was used for the purpose of procedure planning, with the interventionalist first selecting the slice allowing for optimal discrimination of the access route to the neuroforamina for the periradicular infiltration. Planning was performed directly on the consoles of the MDCT system. During the subsequently performed interventional procedure, sequential scanning was performed for surveillance using a foot pedal (intermittent scanning, three axial images per shot).

**Figure 1:**
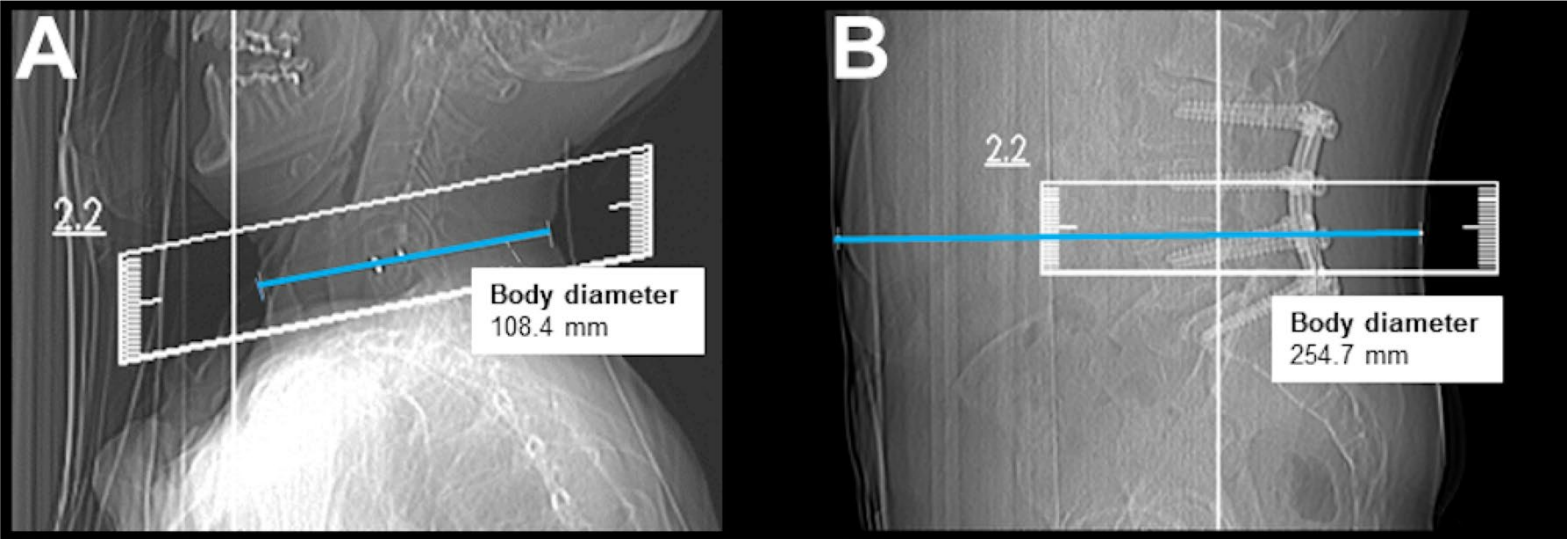
Lateral scouts obtained for a therapeutic C6 nerve root injection in a 58-year-old woman (**A**) and for a diagnostic L4 nerve root injection in a 73-year-old man (**B**). The blue line indicates the anteroposterior body diameter [108.4 mm in (**A**) and 254.7 mm in (**B**)].

Regarding the scanning protocol, the SD scans were acquired with 40 mA and 120 kVp by default. The LD scans were obtained with 20–30 mA and 120 kVp (Table 1). The slice thickness was 1 mm, and images were reconstructed with model-based iterative reconstruction (IMR3; Philips Healthcare, Best, The Netherlands) without further dedicated applications for additional metal artifact reductions. Parameters obtained from SD and LD scanning included the volumetric CT dose index (CTDI_vol_) and dose length product (DLP), the total time (survey scan to last scan acquired during the procedure), number of scans required to perform the periradicular infiltration (periprocedural scans via sequential scanning), and estimates of body diameter. The individual body diameter was measured in the lateral scout scan at the level of the planned intervention and was taken from skin-to-skin surface (Fig. 1)^15^.

**Table 1:** Scanning details and image reconstruction for scanning with standard dose (SD) and low dose (LD).

	SD	LD
Scan increment (in mm)	40.0	
Cycle time (in s)	2.4	
Number of circles	1	
Rotation time (in s)	0.75	
Average scan time (in s)	2.4	
Axial pixel spacing (in mm^2^)	0.39 × 0.39	
Tube voltage (in kVp)	120	120
Tube current (in mA)	40	20–30
Exposure (in mAs)	30	15–20
Slice thickness (axial, in mm)	1	
Image reconstruction	IMR3	
Voxel resolution	Restricted with respect to the fixed collimator width of the detector pixel

### Image evaluation

**Figure 2:**
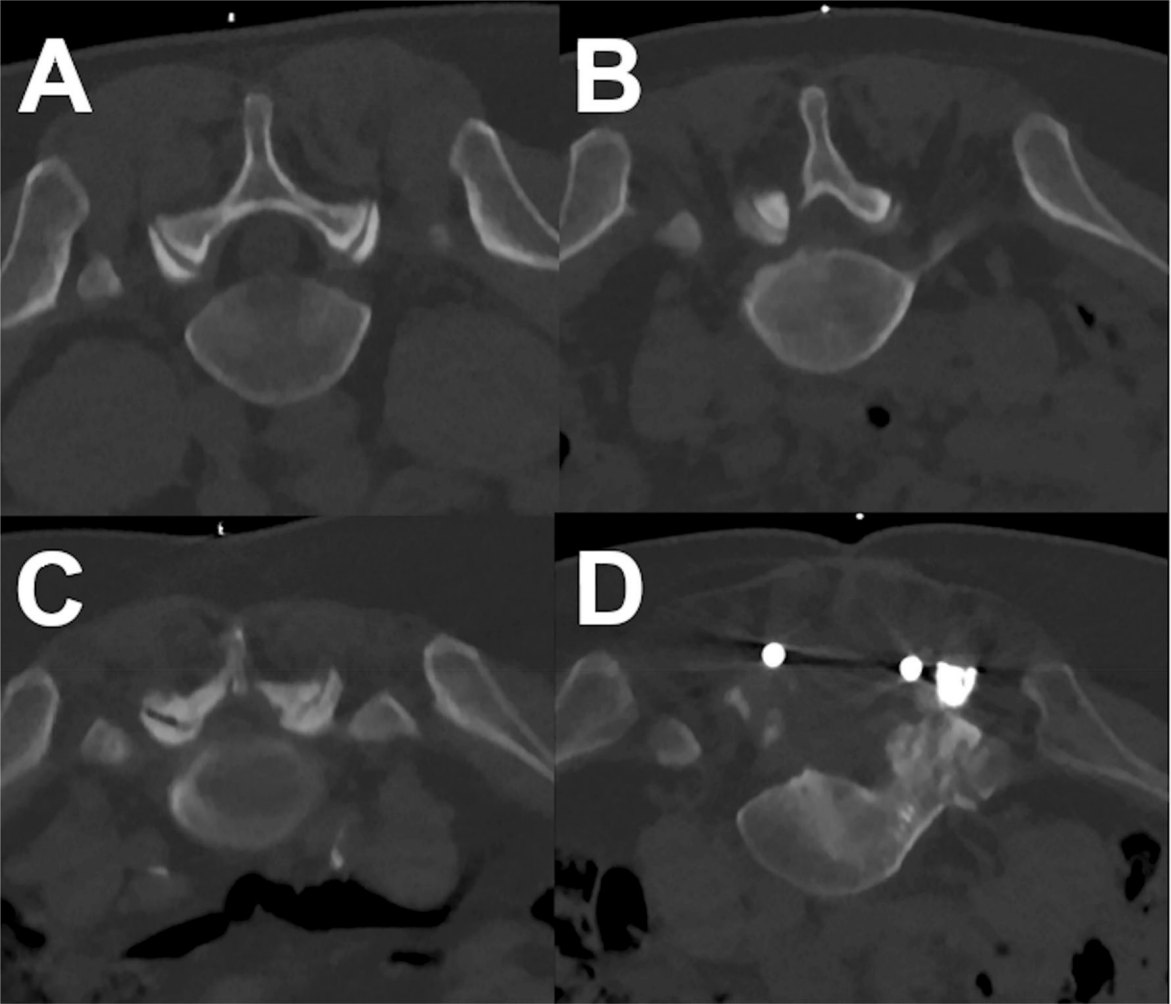
Example for a planning scan for a right-sided L5 nerve root injection in different patients performed with low dose (LD), which were rated with perfect (**A**), good (**B**), medium (**C**), and poor (**D**) overall image quality.

Reconstructed image data of all patients were transferred to PACS and evaluated in the standard PACS viewer (IDS7; Sectra AB, Linköping, Sweden). Image evaluation was performed by two neuroradiologists (9 years of experience in radiology, reader 1 [R1], and 2 years of experience, reader 2 [R2]), who assessed overall image quality, overall artifacts, and image contrast based on 5-point Likert scales (Table 2; Fig. 2). In addition, determination of the nerve root for the planning scan and confidence for intervention planning (based on planning scans) and confidence for intervention guidance (based on the sequential scans during performance of the infiltration) were rated using 3-point Likert scales (Table 2; Fig. 2). The number of grades per evaluated item and their definition were chosen in agreement with previous work on semi-quantitative evaluation of MDCT with LD imaging^30,33,34^.

**Table 2:** Scoring scheme for image analysis.

Item	Score				
	1	2	3	4	5
Overall image quality	Very good to perfect	Good to very good	Medium	Poor	Inadequate
Overall artifacts	None	Minimal	Prominent	Major	Severe
Image contrast	Very good to perfect	Good to very good	Medium	Poor	Inadequate
Determination of nerve root	Possible	Unclear	Not possible	x	x
Confidence for intervention planning (planning scans before the infiltration)	High	Medium	Low	x	x
Confidence for intervention guidance (sequential scans during the infiltration)	High	Medium	Low	x	x

Evaluations were performed after patient pseudonymization, and the readers were strictly blinded to the ratings of each other during evaluations of the SD and LD data. Furthermore, there was an interval of at least 2 weeks between the readings of data of different dose levels, with the order of patient cases being subject to randomization during reading. Information about the location of the planned periradicular infiltration was accessible to both readers, who were, however, not given details about acquisition or dose characteristics (SD vs. LD scans). Readers were allowed to switch between bone and soft tissue windowing during evaluations, and they were able to manually adjust windowing levels if wanted.

**Figure 3:**
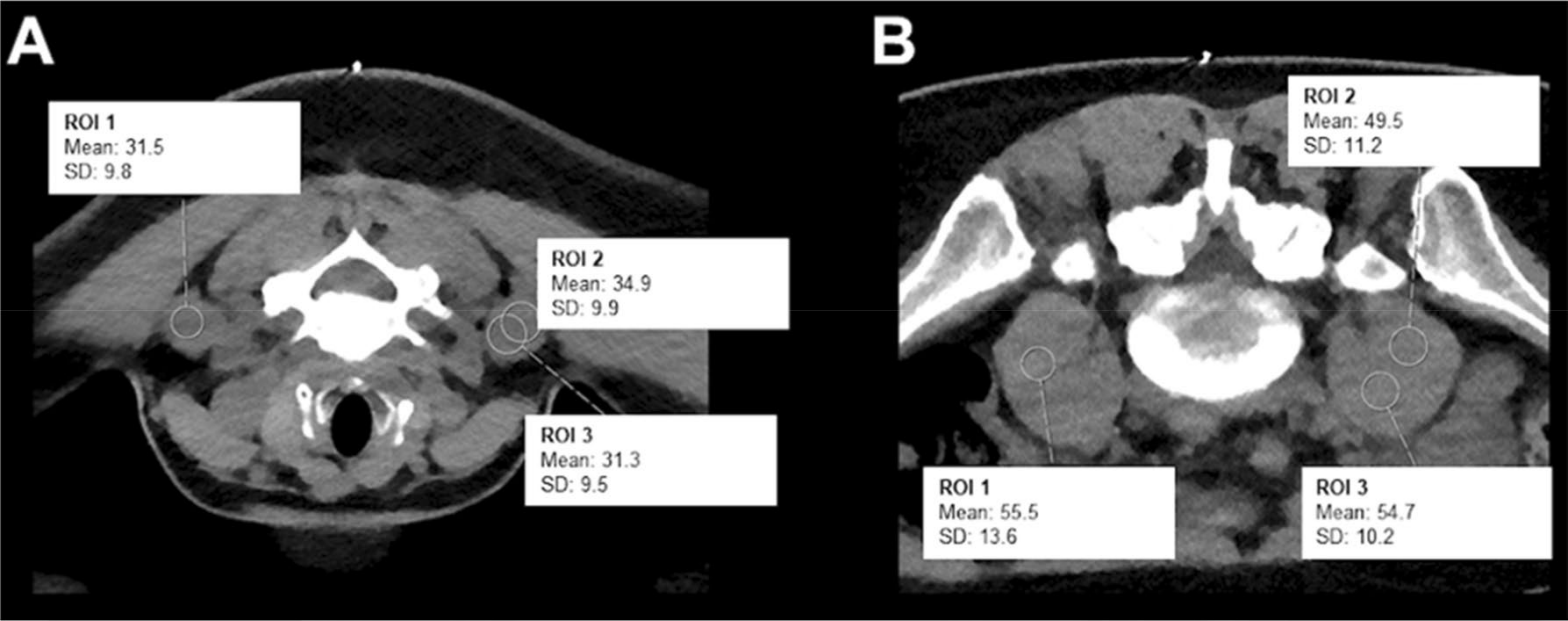
Measurement of image noise for a planned left-sided diagnostic C5 nerve root injection in a 41-year-old woman (**A**) and a planned left-sided L5 nerve root injection in a 34-year-old man (**B**). Scans were acquired with standard dose (SD) in these two exemplary patient cases. Three measurements of cervical or psoas muscle attentuation values were derived from three circular regions of interest (ROIs).

Quantitative evaluation was performed in accordance to literature by measuring image noise on the axial CT planning images at the level of injection by manually placing ~10 mm^2^ circular regions of interest (ROIs) and measuring the standard deviation (StDev) of the attenuation in Hounsfield units (HU) in the psoas muscles for lumbosacral and cervical muscles for cervical interventions^15,35^. Three measurements within either the psoas or cervical muscles at three different regions were performed to obtain a mean value as a surrogate measure for later analysis (Fig. 3). The obtained values of the three ROIs were averaged per patient.

### Statistical analysis

For statistical analyses, GraphPad Prism (version 6.0; GraphPad Software, San Diego, CA, USA) and SPSS (version 20.0; IBM SPSS Statistics for Windows, IBM, Armonk, NY, USA) were used. A *p* value < 0.05 was considered statistically significant.

Descriptive statistics including absolute or relative frequencies, mean, and StDev were calculated for demographics, characteristics of interventions and dose measurements, and for the scores assigned by each reader concerning the single items of image evaluation and attenuation measurements for quantitative image evaluation. Weighted Cohen’s kappa (κ) was calculated to evaluate agreement between readers regarding scorings for overall image quality, overall artifacts, image contrast, determination of nerve root, and confidence for intervention planning as well as for periprocedural guidance of the infiltration. Furthermore, to compare scores of the readers’ image evaluation between scans acquired with SD and LD, Wilcoxon signed-rank tests were conducted for each reader, respectively. Wilcoxon signed-rank tests were also calculated to compare measures of image noise between scans acquired with SD and LD, demographics, and dose characteristics.

### Ethical approval

This retrospective monocentric study involving human participants with a matched pairs design was approved by the ethics committee of the Technical University of Munich and was in accordance with the ethical standards of the institutional research committee and with the Declaration of Helsinki.

### Informed consent

The requirement for written informed consent was waived by the ethics committee of the Technical University of Munich due to the study’s retrospective design.

## Results

### Patient cohort

**Figure 4:**
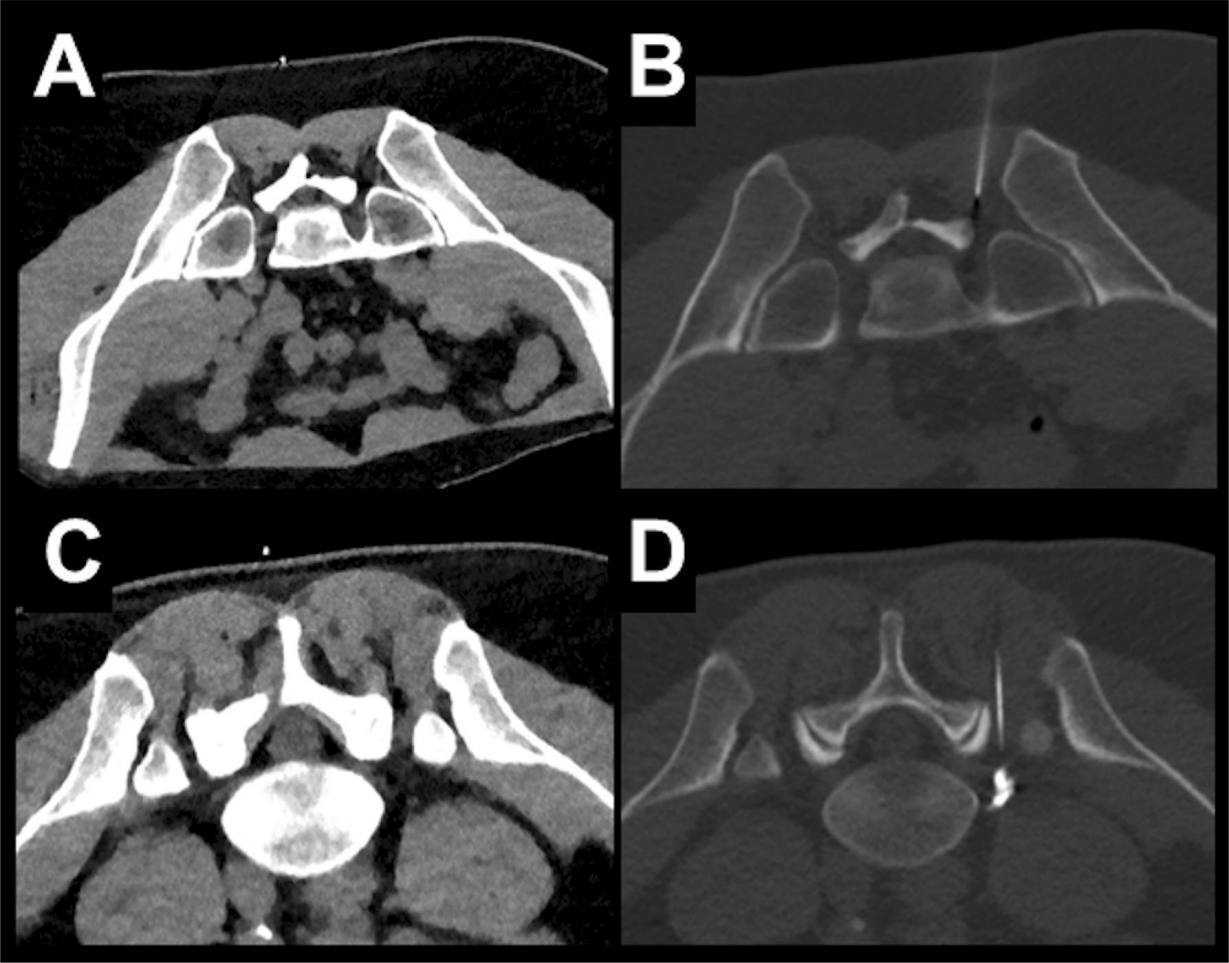
Examples for planning scans and periprocedural guidance scans for a left-sided diagnostic S1 nerve root injection in a 23-year-old male using scanning with standard dose (SD; **A**, **B**) and a left-sided L5 nerve root injection in a 53-year-old male using scanning with low dose (LD; **C**, **D**). The scans were rated with excellent image quality and high confidence for planning and intervention guidance.

**Figure 5:**
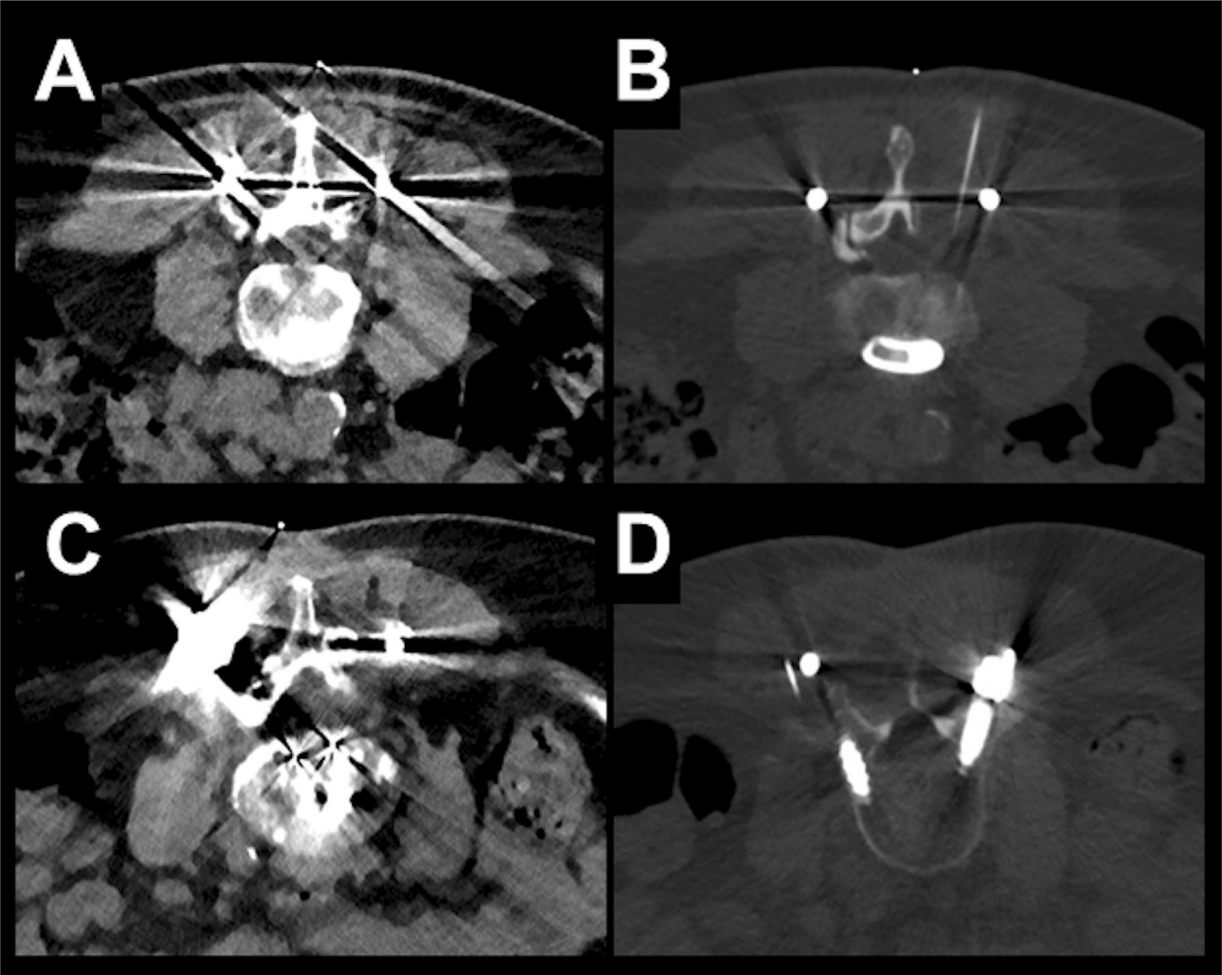
Examples for planning scans and periprocedural guidance scans for a left-sided diagnostic L3 nerve root injection in a 70-year-old male using scanning with standard dose (SD; **A**, **B**) and a right-sided L4 nerve root injection in a 70-year-old female performed with low dose (LD; **C**, **D**). Both patients had undergone dorsal stabilization with metal hardware. The overall image quality was rated as poor for each scan, resulting in low confidence for planning and intervention guidance.

**Figure 6:**
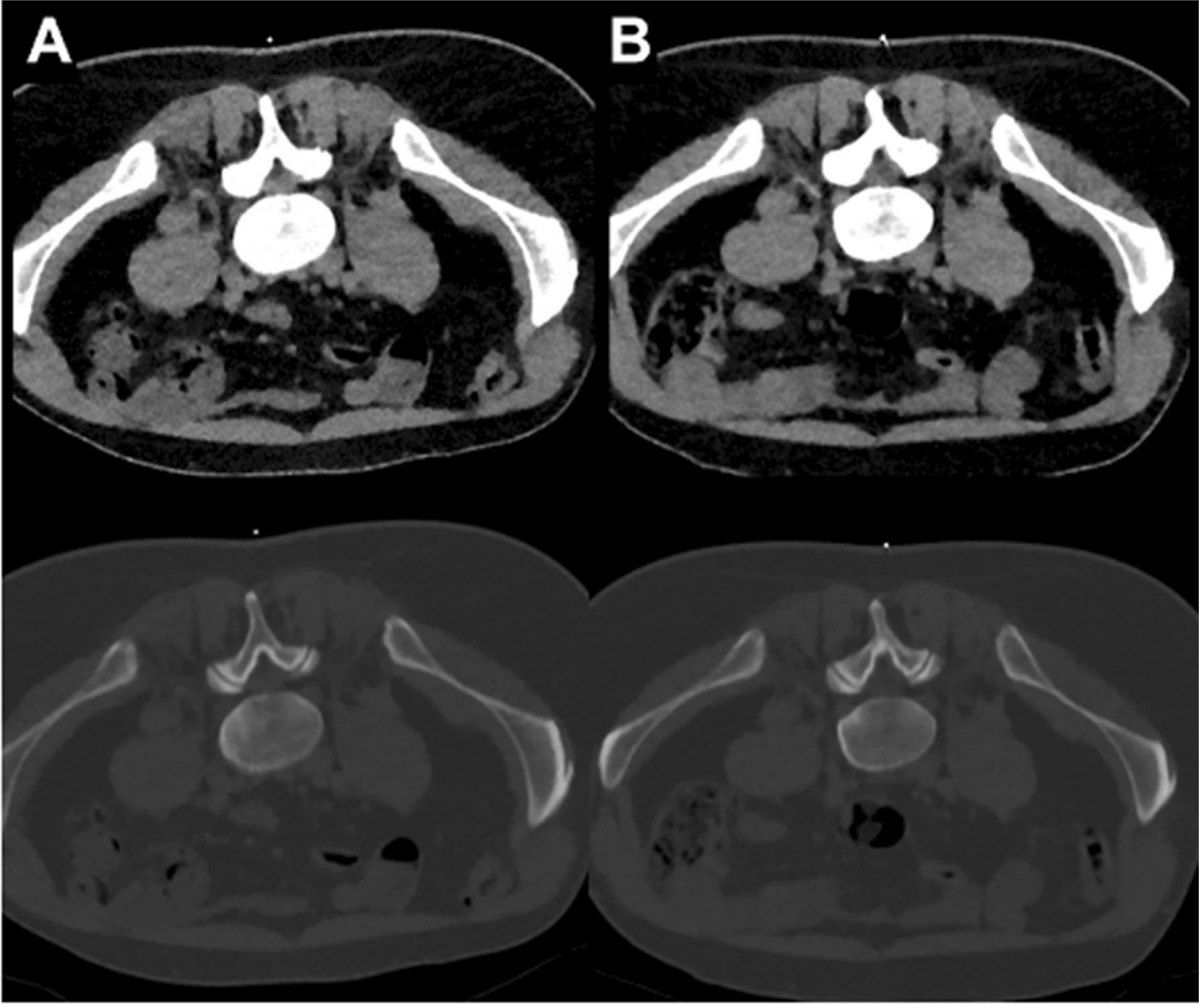
Examples for planning scans (bone and soft tissue windowing) of a right-sided L5 nerve root injection in a 42-year-old male with scanning at low dose (LD; **A**) and scanning with standard dose (SD; **B**). Acquisitions were performed 14 months apart from each other. Both scans were rated with excellent overall image quality and high confidence for intervention planning.

Overall, we identified a total number of 324 eligible subjects (175 cases with LD and 149 cases with SD acquisition). Out of these cases, 204 matched cases were evaluated (102 SD scans and 102 LD scans). According to matching criteria, 51.0% of patients were female in both groups, and 83.3% of periradicular infiltrations were performed at the lumbosacral and 16.7% of periradicular infiltrations were performed at the cervical spine. There were no statistically significant differences between patients for SD versus LD scans regarding age, body diameter, or number of sequential scans needed during performance of the periradicular infiltrations (Table 3). No major periprocedural complications (e.g., bleeding) were reported for any of the periradicular infiltrations. Exemplary patient cases are shown in Figs. 4, 5 and 6.

**Table 3:** Cohort and procedure characteristics.

	SD	LD	*p* value
Age (in years, mean ± StDev)	64.68 ± 14.21	64.14 ± 14.43	0.89
Body diameter on scout image (in cm, mean ± StDev)	21.51 ± 4.64	21.56 ± 4.58	0.64
Number of scans needed during intervention (n)	7.75 ± 5.87	7.94 ± 4.43	0.86
Time needed for the entire procedure (in min, mean ± StDev)	16.62 ± 7.35	14.95 ± 10.92	0.01

### Image evaluation

Overall image quality was scored as good to very good (average Likert score 2) with only minimal artifacts (average Likert score 2) according to both readers, with no statistically significant differences between scores for SD and LD imaging per reader, respectively (*p* > 0.05; Table 4). Similarly, image contrast was rated as very good to perfect (average Likert score 1) by both readers for SD and LD imaging, yet with a statistically significant difference for R2 due to slightly worse scores for LD acquisitions (*p* = 0.04; Table 4).

**Table 4:** Results of image evaluation using Likert scales (mean scores ± standard deviation [StDev]) for evaluations of reader 1 [R1] and reader 2 [R2].

	SD	LD	*p* value
**Overall image quality**			
R1	1.60 ± 0.85	1.54 ± 0.74	0.46
R2	1.59 ± 0.80	1.53 ± 0.82	0.44
Kappa	0.83	0.86	
**Overall artifacts**			
R1	1.51 ± 0.87	1.38 ± 0.72	0.17
R2	1.46 ± 0.80	1.43 ± 0.79	0.65
Kappa	0.87	0.87	
**Image contrast**			
R1	1.30 ± 0.61	1.21 ± 0.49	0.22
R2	1.28 ± 0.49	1.42 ± 0.62	0.04
Kappa	0.73	0.62	
**Determination of nerve root**			
R1	1.13 ± 0.34	1.12 ± 0.35	0.99
R2	1.16 ± 0.37	1.10 ± 0.33	0.21
Kappa	0.72	0.83	
**Confidence for intervention planning**			
R1	1.13 ± 0.34	1.12 ± 0.35	0.99
R2	1.11 ± 0.31	1.10 ± 0.33	0.99
Kappa	0.72	0.83	
**Confidence for intervention procedure**			
R1	1.02 ± 0.14	1.02 ± 0.14	0.99
R2	1.03 ± 0.17	1.02 ± 0.14	0.99
Kappa	0.80	0.49	

Determination of the nerve root was unambiguously possible (average Likert score 1) in almost all patients without a statistically significant difference between SD and LD acquisitions according to both readers (*p* > 0.05; Table 4). Hence, images of SD and LD acquisitions led to high confidence for intervention planning as well as for periprocedural intervention guidance (average Likert score 1) without a statistically significant difference between readers (*p* > 0.05; Table 4). Thus, no periradicular infiltration had to be aborted due to impaired confidence for the performing neuroradiologist. Between readers, agreement for scores of image evaluations was at least substantial (κ ≥ 0.62), except for confidence in intervention planning for LD scans with moderate agreement between readers (κ = 0.49; Table 4).

According to quantitative image evaluation, noise in imaging data was comparable between scans acquired with SD and LD schemes, showing no statistically significant differences between protocols (13.13 ± 3.66 HU vs. 13.37 ± 4.08 HU; *p* = 0.85).

### Radiation doses

**Figure 7:**
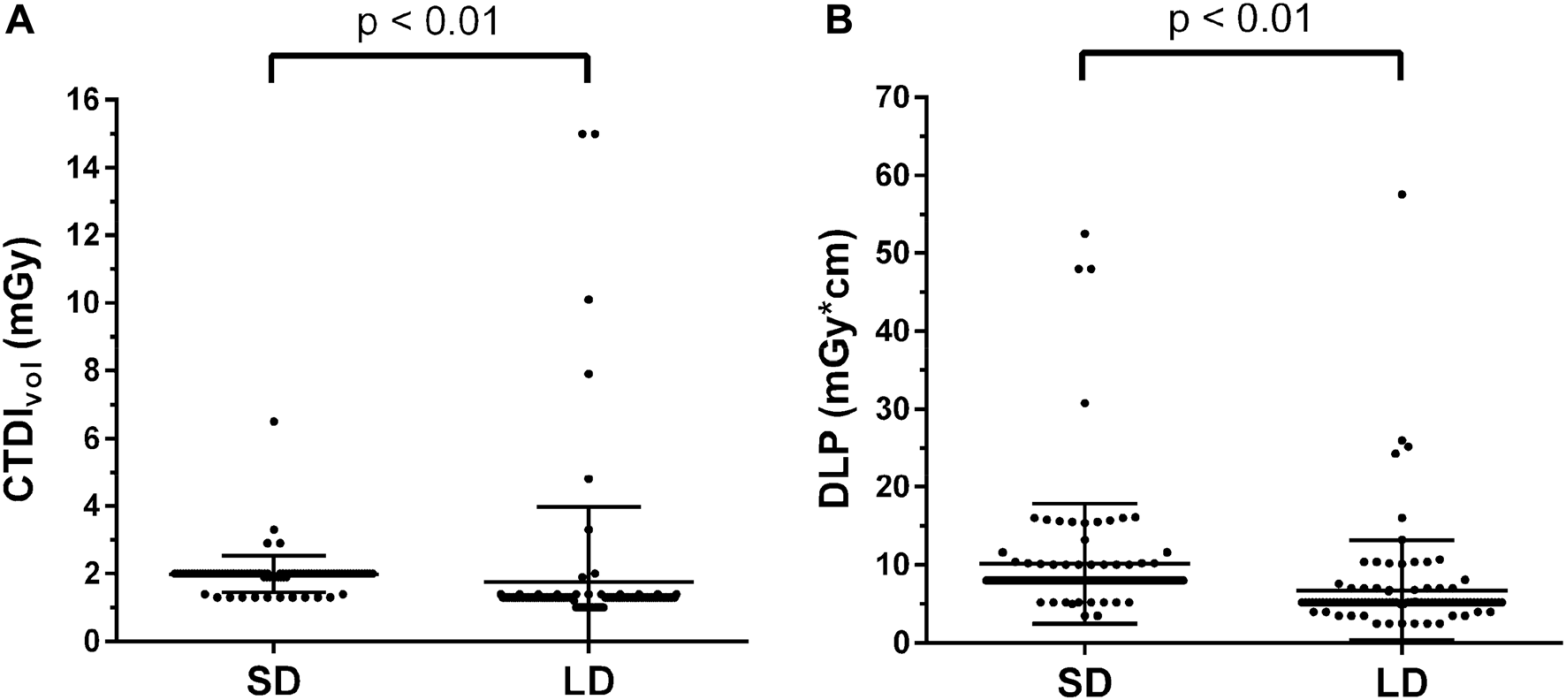
Scatter plots with horizontal lines for mean ± standard deviation (StDev) for the volumetric computed tomography dose index (CTDI_vol_ in mGy; **A**) and dose length product (DLP in mGy*cm; **B**), considering scanning with standard dose (SD) and low dose (LD).

The CTDI_vol_ was statistically significantly lower for LD scans compared to SD scans for the planning scans (1.76 ± 2.21 mGy vs. 1.99 ± 0.54 mGy; *p* < 0.01; Fig. 7). Similarly, the DLP was statistically significantly lower for LD scans acquired for intervention planning, showing a reduction of 33.5% on average when comparing SD to LD scans (6.75 ± 6.43 mGy*cm vs. 10.16 ± 7.70 mGy*cm; *p* < 0.01; Fig. 7).

## Discussion

Lowering the tube current for MDCT can be a simple and effective method for reducing radiation exposure to both the patient and interventionalist regarding periradicular infiltrations. In our study, we showed that dose reduction for particularly planning scans of periradicular lumbosacral and cervical infiltrations with MDCT is feasible and can be performed without clinically relevant drawbacks regarding image quality or confidence for planning or conducting the infiltrations. The CTDI_vol_ was statistically significantly lower for LD scans compared to SD scans while image noise was comparable between SD and LD scans. Overall image quality and image contrast were scored very good for both protocols, which made determination of the nerve root possible in almost all patients, leading to high confidence for intervention planning and periprocedural intervention guidance.

It is well known that the use of diagnostic CT has distinctly increased over the past years^18,36^. However, not only the diagnostic use but also the use of CT in interventional radiology for image guidance and navigation purposes has increased over time, which is mostly due to an increasing trend to minimally invasive medicine^14,37^. This is a development that comes at cost of higher radiation exposure and, as a consequence, at potentially increased estimated cancer risk ratios for patients and interventionalists exposed to CT^16–21^. Hence, it should be the aim to perform these CT scans with the lowest reasonable radiation exposure but, at the same time, without losing sight of the clinical usefulness of generated images with respect to the “as low as reasonably achievable” (ALARA) principle^38,39^.

Periradicular infiltrations make up a major proportion among the increasing numbers of image-guided interventions that are more and more frequently performed using MDCT for initial procedure planning on the one side and surveillance of the procedure on the other side. As this kind of intervention is widely used, previous work exists that aimed to lower radiation exposure for periradicular infiltrations using CT scanning, combined with different approaches for restricting radiation dose exposure that mostly included iterative software for image reconstructions, tube current modulation, and replacing the planning scan with a single-spot fluoroscopy image^15,35,40–42^. Although obvious measures reducing radiation exposure focus on the fluoroscopic or periprocedural component of the intervention, a major part of the radiation exposure is often delivered during the preprocedural planning scans^31^. Specifically, one previous study investigated and successfully applied LD imaging for periradicular lumbosacral infiltrations with 100 kV and a tube current–time product of only 5 mAs in patients with a body mass index (BMI) lower than 30 kg/m^2^ without constraints for safety or efficiency^4^. Furthermore, this study group investigated the use of and need for a hybrid reconstruction software compared to filtered back projection (FBP) in CT-guided periradicular infiltration therapy at the lumbar spine with the same LD protocol^40^. They evaluated both types of reconstruction algorithms regarding conspicuity of anatomical and instrumental features important for ensuring patient safety and measured image noise as a quantitative marker of image quality^4,40^. They concluded that in spite of a marked reduction of image noise, hybrid reconstruction was not necessary for adequate image quality^4,40^. Other studies focusing on reducing radiation exposure by reducing the tube current only in the planning scans were performed with mostly higher parameters. Amrhein et al. studied the effect on lumbar spine pain injections by reducing the dose parameters from 120 kV and 110–440 mA to 120 kV and 50–100 mA depending on the body diameter^15^. Shepherd et al. investigated the effect for cervical injections from 104 ± 68.2 mA to 16.7 ± 11.2 mA and from 120 kV with 37.7 ± 29.0 mA to 120 kV with 11.7 ± 4.9 mA for lumbar injections.

Furthermore, a recent study systematically analyzed various items of image quality and confidence for intervention planning of lumbosacral periradicular infiltrations considering simulations of LD imaging by virtually lowering the tube currents step by step (50 mAs down to 1 mAs; 50% down to 1% of the tube current of original scanning) based on raw data taken from the same MDCT system for the whole cohort^30^. The study concluded that in this virtual simulation setting a reduction of the tube current for planning of lumbosacral periradicular infiltrations in interventional scanning by MDCT is feasible and can therefore be performed with only 10% of original tube current (equal to 10 mAs) without clinically relevant drawbacks regarding confidence for intervention planning^30^. Yet, at the same time, the level of regularization of iterative reconstruction plays a considerable role for image quality and contrast, and the results of a simulation study might not necessarily be generalizable to the in-vivo clinical setup^30^.

Related to this study’s retrospective design, the BMI was not routinely available for the patients of our cohort. We therefore measured the anteroposterior body diameter to consider the individual body habitus of the patients included. Although using BMI calculations is the approach more frequently chosen, several researchers favor the use of the anteroposterior body diameter in the same or similar manner as performed in our study. On the one hand, it correlates with the distance of the pathway traversed by the x-ray beam and, on the other hand, it is easy to measure on the scout image before the procedure, thus also seamlessly available for retrospective study designs. This concept of tailoring CT dose settings to patient size seems reasonable as body size-specific protocols are used in CT imaging in pediatric radiology^43^. A previous study compared tube current and procedure time for lumbar spine CT-guided selective nerve root blocks, and afterwards correlated image quality to patient diameter and tube current^27^. The authors concluded that the anteroposterior diameter had the greatest influence on image quality, thus they recommended a tube current of 40 mA or less for an anteroposterior body diameter of < 30 cm^27^.

There are some limitations to this study. First, we used a retrospective setup performed at a single academic institution, implicating that periradicular infiltrations were conducted by different neuroradiolgists with different education levels. As a consequence, there is a potential bias for radiation dose exposure as well as procedural time and number of scans needed during intervention that is inherent to the study design. Second, lowering of radiation exposure was reached by reducing the tube current in combination with model-based iterative reconstruction, but without evaluating other modern approaches to limit radiation exposure, such as sparse sampling^34,44^. Yet, sparse sampling during image acquisition has not yet been implemented in commercially available MDCT systems, but may become of general interest in the near future.

## Conclusion

We demonstrated that a LD imaging protocol combined with advanced image reconstruction for MDCT scanning during planning of periradicular infiltrations is a viable option as we could show that radiation doses from CT-guided spinal procedures decreased without significantly affecting the image quality or confidence for intervention planning and performance. Particularly given the frequent repeated procedures in case of treatment success with pain reduction for periradicular infiltrations, lowering of radiation exposure to a minimum is crucial. We therefore encourage other centers to consider LD imaging protocols combined with model-based iterative reconstruction as an option for MDCT-guided periradicular infiltrations in diagnostic and therapeutic pain management scenarios.
